# Diagnostic agreement and limitations of the Banff automated system in kidney transplant biopsies

**DOI:** 10.3389/fneph.2025.1645280

**Published:** 2025-11-20

**Authors:** Jun Matsushita, Toshihito Hirai, Tomokazu Shimizu, Yu Kijima, Kohei Unagami, Masaaki Yanishi, Hidefumi Kinoshita, Toshio Takagi, Hideki Ishida

**Affiliations:** 1 Department of Organ Transplant Medicine, Tokyo Women’s Medical University Hospital, Tokyo, Japan; 2 Department of Urology, Kansai Medical University, Osaka, Japan; 3 Department of Urology, Tokyo Women’s Medical University Hospital, Tokyo, Japan

**Keywords:** kidney transplantation, Banff automated diagnosis system, ABO incompatible kidney transplant, antibody mediated rejection, T-cell mediated rejection

## Abstract

The Banff classification for renal allograft rejection has evolved over time, increasing in complexity. For non-pathologists conducting retrospective studies, assigning Banff diagnostic categories across different eras presents a significant challenge. The Automated Diagnosis System (ADS) is a publicly available web-based tool designed to standardize Banff category diagnoses based on Banff scoring. We retrospectively evaluated ADS using 1,071 kidney biopsy results from 544 transplant recipients, including 146 ABO-incompatible cases performed at our institution. Overall concordance between ADS and pathologists was 69.8%, with high agreement in non-rejection (97.4%) and rejection (86.3%) cases. Among rejection cases, discrepancies were noted in 27 antibody-mediated rejection (AMR) and 22 T cell–mediated rejection (TCMR) cases. Discrepancies were frequently observed in AMR following ABO-incompatible transplantation and in chronic TCMR, highlighting challenges in standardizing these categories. Despite these limitations, ADS demonstrated acceptable concordance and potential utility for promoting global standardization in rejection diagnosis.

## Introduction

Antibody mediated rejection (AMR) is a critical factor influencing the prognosis of kidney allografts (1) but its diagnosis remains complex. Initially defined at the 2001 Banff Conference as a condition characterized by microvascular inflammation (MVI), C4d deposition, and the presence of donor-specific antibodies (DSA) in the serum, the diagnostic criteria for AMR have undergone multiple revisions over the years (1, 2). Notably, C4d-negative AMR has been newly proposed, while ABO-incompatible transplants often demonstrate C4d positivity—defined as diffuse linear staining of peritubular capillaries (PTCs) for C4d in ≥50% of cortical PTCs—regardless of the presence of rejection (3, 4). These complexities make accurate diagnosis challenging, particularly for non-specialists in transplant pathology.

To address these challenges, Yoo et al. developed a web-based diagnostic tool capable of providing AMR diagnoses aligned with the latest Banff criteria (2019) by inputting Banff scores (5). This automated diagnosis system (ADS), free from human bias, has been reported to predict allograft outcomes more accurately than traditional pathologist-based diagnoses, highlighting the potential benefits of diagnostic standardization (6). However, the validation of this tool has predominantly relied on datasets in which 70–80% of cases were deceased donor transplants, while ABO-incompatible transplants—accounting for only 2–4% of the cohort—were underrepresented.

Given the high prevalence of ABO-incompatible transplants in Japan, there is a need to evaluate the applicability of this system in an international context. This study aims to validate the performance of the ADS in a Japanese kidney transplant cohort with a significant proportion of ABO-incompatible cases.

## Methods

This study was approved by the Institutional Review Board (IRB) of Tokyo Women’s Medical University (approval number: 2023-0033), and the procedures followed were in accordance with the ethical standards of the local IRB and with the Helsinki Declaration of 1975, as revised in 2013. Pathological data were retrieved from the database of 1,071 allograft kidney biopsies from 544 recipients underwent kidney transplant at Tokyo Women’s Medical University between January 2017 and July 2022, including 146 cases with ABO incompatibility. Banff scores and pathological diagnosis were determined by professional pathologist according to the Banff criteria at each time point. In samples which had more than two diagnoses, the most dominant rejection feature was selected as primary rejection category. The ADS processed each case using input data, including Banff lesion scores, C4d immunofluorescent staining results, and DSA status. Except the presence or absence of thrombotic microangiopathy (TMA), non-rejection-related diagnoses, such as IgA nephropathy and focal segmental glomerulosclerosis (FSGS), were not incorporated for the ADS processing. All cases were classified into one of five AMR categories though the ADS: active AMR (A-AMR), chronic active AMR (CA-AMR), equivocal for diagnosis of AMR, C4d staining without evidence of rejection that is classified as linear staining in ≥50% of cortical PTCs with no histologic evidence of acute tissue injury consistent with AMR, and no evidence of AMR. T-cell mediated rejection (TCMR) were also classified into acute TCMR (A-TCMR) and chronic active TCMR (CA-TCMR). The primary rejection category was selected according to the following priority: A-AMR (including equivocal for diagnosis), CA-AMR, A-TCMR, C-TCMR, chronic-AMR inactive, borderline change for TCMR (BC), interstitial fibrosis and tubular atrophy (IFTA), C4d staining without evidence of rejection, and no evidence of rejection.

## Results

**Figure 1:**
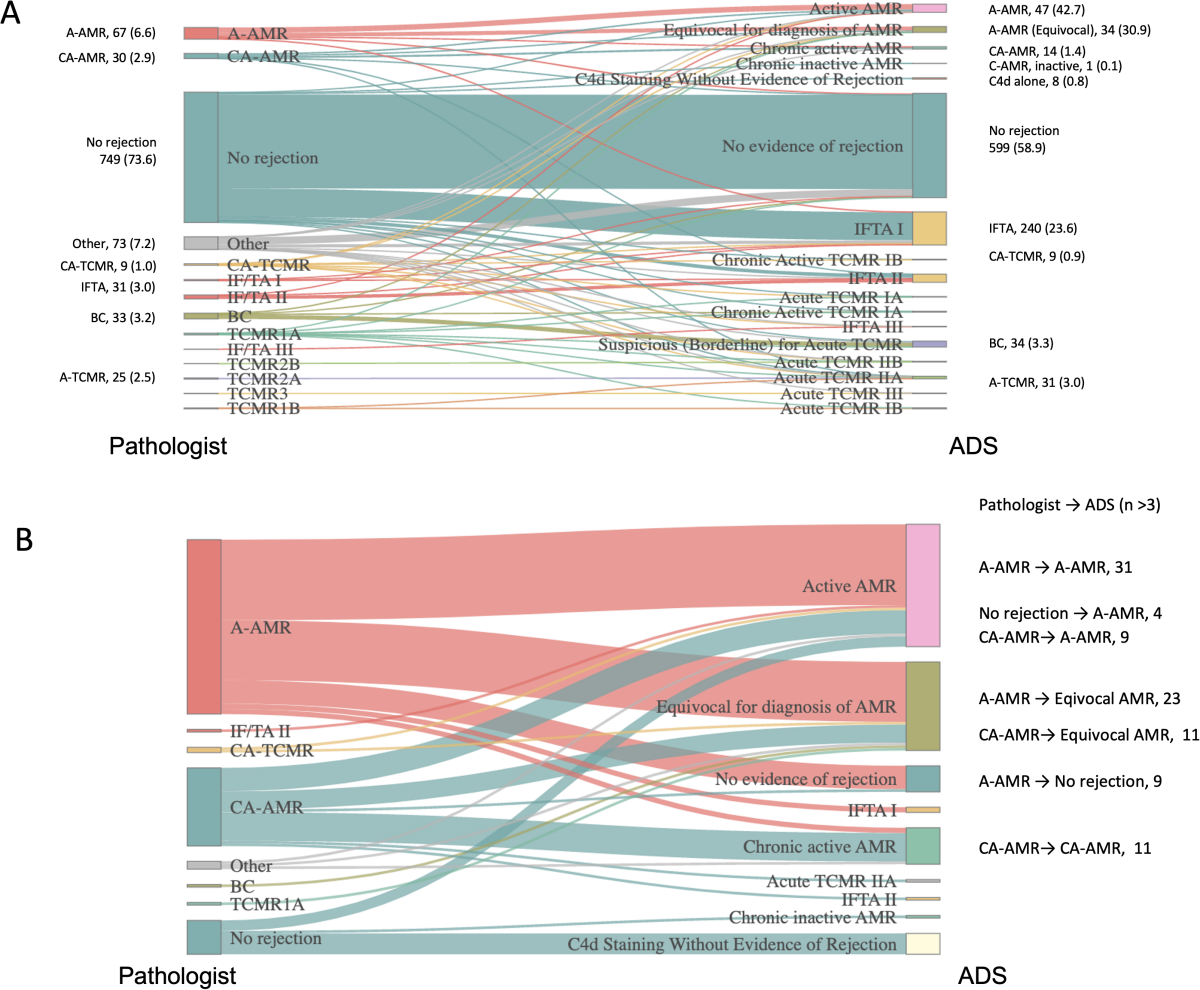
Sanky diagram showing relationship between pathologist- and automated diagnosis system-diagnoses. **(A)** Overall comparison. **(B)** Focused on results related to antibody mediated rejection. A-AMR, Active Antibody Mediated Rejection: CA-AMR, Chronic Active Antibody Mediated Rejection; A-TCMR, Acute T Cell Mediated Rejection I – III; CA-TCMR, Chronic active T cell Mediated Rejection; BC, Borderline Change; IFTA, Interstitial Fibrosis and Tubular Atrophy I – III.

**Figure 1** presents a Sankey diagram illustrating the relationship between pathologists’ diagnoses and ADS-based reclassification of 1071 biopsy results. Among these, ADS diagnoses were concordant with pathologists’ diagnoses in 747 cases, yielding an overall agreement rate of 73.45%. Among the 886 cases classified as “No Rejection” by pathologists (including 31 cases of IFTA, 33 cases of BC, and 73 cases with other diagnoses), 863 cases were also categorized as “No Rejection” by ADS (including 8 cases of C4d staining without evidence of rejection; 234 cases of IFTA; and 33 cases of BC). While pathologists tend to avoid using mild IFTA as the final diagnosis, the agreement rate for non-rejection cases was adequately high (97.4%), indicating minimal discrepancies.

On the other hand, among the 131 cases diagnosed as rejection by pathologists, 113 cases were also classified as rejection by ADS, resulting in an agreement rate of 86.3%. However, complete concordance in rejection type was observed in only 87 cases (66.4%). Furthermore, 18 cases diagnosed as rejection by pathologists were re-classified as non-rejection by ADS (A-AMR: 11 cases, CA-AMR: 2 cases, A-TCMR: 2 cases, CA-TCMR: 3 cases). Conversely, 23 cases diagnosed as no-rejection by pathologists were re-categorized as rejection by ADS (A-AMR: 8 cases, CA-AMR: 2 cases, A-TCMR: 6 cases, CA-TCMR: 7 cases).

**Table 1** lists the cases with diagnostic discrepancies for AMR. Fourteen cases diagnosed as AMR by pathologists were not classified as AMR by ADS. The majority of these cases did not meet the threshold for the MVI score. Although C4d deposition was observed in 11 cases, its diagnostic significance was uncertain in 7 cases due to ABO-incompatible transplantation. Some cases included follow-up biopsies after rejection treatment, where AMR diagnosis was made based on clinical history rather than Banff scoring alone. Conversely, 13 cases classified as AMR by ADS had different pathological diagnoses. Nine of these were “for-cause biopsies” refer to biopsies performed when rejection was suspected based on elevated creatinine levels, proteinuria, or other clinical findings, suggesting that pathologists may have exercised caution in determining treatment interventions. Additionally, cases of recurrent glomerulonephritis and BK nephropathy, which were not influenced by the Banff scores input in ADS, were included. Although not examined in this study, ADS allows for the input of diagnoses other than rejection and previous biopsy findings, suggesting that incorporating detailed clinical and histopathological information could help resolve such discrepancies in real-world applications.

**Table 1:** AMRs with different diagnosis between pathologists and ADS.

ID	Bx year	Bx type	Day after Tx	ABO	HLA DSA	t	i	v	g	ptc	C4d	cg	cv	ptcbm	Pathologist Diagnosis	ADS Diagnosis
AMR Diagnosed by Pathologist Alone																
104347	2018	Protocol	359	compatible	–	0	0	1	0	1	2	0	1	1	CA-AMR	A-TCMRIIA
104461	2018	Protocol	347	incompatible	–	0	0	0	1	0	0	0	0	0	A-AMR	No Rejection
104529	2019	Protocol	359	incompatible	–	0	0	0	1	0	2	0	0	0	A-AMR	No Rejection
104601	2019	Protocol	365	compatible	–	1	0	0	0	2	1	0	0	0	A-AMR	IFTA
104797	2019	Follow up	58	incompatible	–	0	0	0	0	1	2	2	3	0	CA-AMR	IFTA
104797	2019	Follow up	24	incompatible	–	0	0	0	0	1	0	0	3	0	A-AMR	No Rejection
104949	2020	For cause	12	incompatible	–	1	0	0	0	1	2	0	0	0	A-AMR	No Rejection
105058	2021	Protocol	105	incompatible	–	0	0	0	1	0	2	0	0	0	A-AMR	No Rejection
105139	2021	Protocol	97	incompatible	–	0	0	0	0	2	1	0	0	0	A-AMR	IFTA
105178	2022	Protocol	115	compatible	+	0	0	0	1	0	1	0	0	0	A-AMR	No Rejection
105182	2022	Protocol	100	incompatible	–	0	0	0	0	1	3	0	0	0	A-AMR	No Rejection
105190	2022	Protocol	89	incompatible	–	0	0	0	1	0	3	0	0	0	A-AMR	No Rejection
105194	2022	Protocol	108	compatible	–	0	0	0	1	0	1	0	0	0	CA-AMR	No Rejection
105237	2022	For cause	6	compatible	+	0	1	0	1	0	0	0	0	0	A-AMR	No Rejection
AMR Diagnosed by ADS Alone																
104309	2017	For cause	113	compatible	–	2	0	0	1	1	0	0	0	0	BC	A-AMR
104431	2017	For cause	1092	incompatible	–	0	0	0	1	1	2	0	0	0	PGNMID recurrence	A-AMR
104488	2018	For cause	870	compatible	–	3	0	0	0	2	0	0	0	0	FSGS recurrence	A-AMR
104553	2018	Protocol	380	compatible	–	0	0	0	1	0	2	0	0	0	No rejection	A-AMR
104580	2018	Protocol	86	compatible	+	0	0	0	0	2	0	0	0	0	No rejection	A-AMR
104580	2018	For cause	16	compatible	+	0	0	0	0	2	1	0	0	0	Toxic tubulopathy	A-AMR
104805	2019	For cause	1083	compatible	–	1	0	0	0	2	2	0	0	1	CA-TCMR	A-AMR
104888	2019	For cause	677	compatible	–	0	0	0	1	2	0	0	0	0	BKV nephropathy	A-AMR
104925	2020	For cause	28	compatible	–	2	0	0	0	2	0	0	0	0	IF/TA	A-AMR
105064	2021	Protocol	497	compatible	–	0	0	0	0	2	0	0	0	0	No rejection	CA-AMR
105128	2021	Follow up	23	incompatible	+	0	0	0	2	0	2	1	0	0	TMA	CA-AMR
105149	2021	For cause	21	compatible	–	0	0	0	2	1	2	0	0	0	FSGS recurrence	A-AMR
105181	2021	For cause	50	compatible	–	1	1	0	2	2	1	0	0	0	A-TCMR 1A	A-AMR

A-AMR, active antibody mediated rejection; A-TCMR, active T cell mediated rejection; ADS, automated diagnosing system; BC, borderline change for TCMR; BKV, BK virus; Bx, biopsy; CA-TCMR, chronic active TCMR; CA-AMR, chronic AMR; DSA, donor specific antibody; FSGS, focal segmental glomerulosclerosis; IFTA, interstitial fibrosis and tubular atrophy; PGNMID, Proliferative glomerulonephritis with monoclonal IgG deposits; TMA, thrombotic microangiopathy; Tx, Transplantation.

Regarding TCMR, 8 cases diagnosed as TCMR by pathologists were not classified as TCMR by ADS, while 14 cases were classified as TCMR by ADS but not by pathologists (**Table 2**). Notably, discrepancies were observed in the diagnosis of CA-TCMR. The classification of CA-TCMR was introduced in Banff 2017, requiring independent evaluation of tubulitis in preserved cortex and cortical IFTA regions (7). Distinguishing CA-TCMR from the mixture of BC or TCMR grade IA/IB and IFTA based solely on Banff scores is challenging, highlighting the need for further discussions to standardize its diagnosis.

**Table 2:** TCMRs with different diagnosis between pathologists and ADS.

ID	Bx year	Bx type	Day after Tx	t	t-IFTA	i	i-IFTA	v	g	ptc	C4d	cg	cv	ci	ct	Pathologist Diagnosis	ADS Diagnosis
TMR Diagnosed by Pathologist Alone																	
104805	2022	Protocol	1083	1	3	0	2	0	0	2	2	0	0	3	3	CA-TCMR	A-AMR
104429	2018	For cause	361	0	–	0	1	0	0	0	0	0	0	0	0	A-TCMR	No rejection
104441	2021	Protocol	1435	0	0	1	3	0	0	2	0	0	0	3	3	CA-TCMR	IFTA
104597	2019	Follow up	178	2	–	1	1	0	0	1	1	0	0	1	1	A-TCMR	BC
104888	2021	For cause	677	0	3	0	2	0	1	2	0	0	0	3	3	CA-TCMR	A-AMR
104962	2021	Protocol	431	0	1	0	1	0	0	0	0	0	0	1	1	CA-TCMR	IFTA
104971	2020	Protocol	88	0	3	0	2	0	0	2	3	0	0	1	1	CA-TCMR	IFTA
105181	2022	Protocol	50	1	0	1	1	0	2	2	1	0	0	2	2	A-TCMR	A-AMR
TMR Diagnosed by ADS Alone																	
105165	2022	Protocol	97	2	0	0	3	0	0	2	0	0	0	0	0	BC	CA-TCMR
105168	2022	Protocol	176	2	1	1	2	0	0	2	0	0	0	2	2	BC	CA-TCMR
104347	2018	Protocol	359	0	–	0	–	1	0	0	1	0	1	0	0	CA-AMR	A-TCMR
104420	2019	For cause	649	2	–	0	2	0	0	2	0	0	0	0	0	No rejection	CA-TCMR
104661	2019	Protocol	373	2	–	1	2	0	0	0	0	0	0	1	1	No rejection	CA-TCMR
104336	2018	For cause	365	3	–	0	2	0	0	2	0	0	0	2	2	No rejection	CA-TCMR
104851	2020	For cause	256	0	3	0	2	0	0	2	0	0	0	2	2	No rejection	CA-TCMR
105052	2021	For cause	206	3	0	1	2	0	0	2	1	0	0	2	2	No rejection	CA-TCMR
104395	2017	Protocol	114	1	–	0	–	1	0	1	3	0	–	0	0	No rejection	A-TCMR
104396	2017	Protocol	85	2	–	2	–	0	0	2	1	0	–	1	1	No rejection	A-TCMR
104866	2020	Protocol	368	0	0	0	0	1	0	1	0	0	0	1	1	No rejection	A-TCMR
104347	2017	Protocol	109	0	–	0	–	1	0	0	1	0	1	0	0	Other	A-TCMR
105060	2021	Protocol	360	0	0	0	0	2	1	0	0	0	0	0	0	Other	A-TCMR
105192	2022	For cause	16	0	–	1	0	3	1	0	2	0	0	0	0	TMA	A-TCMR

A-AMR, active antibody mediated rejection; A-TCMR, active T cell mediated rejection; ADS, automated diagnosing system; BC, borderline change for TCMR; Bx, biopsy, CA-AMR, chronic active AMR; CA-TCMR, chronic active TCMR; IFTA, interstitial fibrosis and tubular atrophy; TMA, thrombotic microangiopathy; Tx, Transplantation.

## Discussion

Overall, ADS showed substantial concordance with pathologists’ diagnoses. However, notable discrepancies in AMR for ABO-incompatible grafts and CA-TCMR highlight ongoing challenges in standardizing these categories. These discrepancies do not necessarily indicate a fundamental flaw in the ADS, but rather reflect the complex and evolving nature of the Banff Classification, as well as the critical role of clinical context in biopsy interpretation. As Van Loon et al. have noted (8), the Banff schema has become increasingly intricate, and pathologists often reach different conclusions by incorporating prior biopsy findings, treatment responses, and clinical impressions—elements not currently captured by the ADS in this study. While the ADS allows entry of “Other diagnosis” inputs in addition to Banff inputs, these appear only as supplementary information in the report and do not directly influence the Banff classification rejection diagnosis logic. In fact, among the 49 cases in which the ADS diagnosis diverged from that of the pathologist in **Tables 1** and **2**, 22 cases (44.9%) were for-cause biopsies prompted by clinical findings such as proteinuria or elevated serum creatinine, or follow-up biopsies performed after treatment for rejection. This underscores the distinction between rule-based classification and clinically integrated diagnosis, highlighting the ADS’s value as a standardized reference tool rather than a substitute for expert judgment.

ABO-incompatible transplantation is frequently performed in Asian countries such as Japan and Korea, where donor shortages persist, but it is not widely practiced in many international centers, and thus limited data are available globally (8, 9). In a previous study, C4d positivity in peritubular capillaries was observed in 94% (diffuse in 66%) of protocol biopsies without any association with ABMR (10), indicating that the diagnostic value of C4d positivity in the setting of ABO-incompatible transplantation remains debated. While the ADS allows entry of ABO incompatibility as an input, the actual number of ABO-incompatible transplants included in the dataset used to develop the algorithm is unknown, and challenges remain regarding its incorporation into the AMR diagnostic algorithm. In our study, 282 of the 1,017 biopsy specimens (27.7%) were from ABO-incompatible kidney transplants. This proportion underscores the unique clinical context of regions such as Japan, where ABO-incompatible transplantation is common, and highlights important considerations for the practical use of the ADS in these settings.

In the current study, a substantial number of discrepancies between ADS and pathologist diagnoses were also observed for CA-TCMR. The diagnosis of CA-TCMR, introduced in Banff 2017, requires nuanced interpretation of tubulitis in areas with and without interstitial fibrosis. Although the ADS incorporates this distinction into its pseudocode, it is likely that technical limitations remain in the algorithm’s ability to fully replicate the subtle interpretive judgments made by experienced pathologists.

As a limitation, we chose a case-by-case approach to discrepancy analysis rather than a wholesale reclassification of all biopsies using the Banff 2019 criteria. In reviewing detailed Banff scores for discrepant cases, we found that differences arose not only from evolving Banff definitions, but also from clinical history, treatment effects, and the controversial nature of AMR diagnosis in ABO-incompatible transplantation—all of which can significantly influence pathologists’ interpretations.

In clinical practice, diagnoses are informed not only by Banff scores but also by histological nuances, longitudinal clinical information, and treatment history. Thus, cautious interpretation of ADS output is warranted. Nevertheless, ADS offers a practical tool for transplant clinicians, especially non-pathologists, by facilitating Banff-based classification in retrospective and multicenter studies. Its role in reducing inter-institutional variability is particularly valuable. Recently, Banff criteria has been updated (11). The major updates include the concept of microvascular inflammation (MVI); e.g. “MVI, DSA-negative, C4d-negative” are no longer considered AMR, MVI below threshold, C4d- but DSA+ cases are deemed “probable AMR”. Future updates to align with the latest Banff iterations are anticipated.

## Data Availability

The raw data supporting the conclusions of this article will be made available by the authors, without undue reservation.
